# Effectiveness of intraosseous access during resuscitation: a retrospective cohort study

**DOI:** 10.1186/s12873-024-01103-w

**Published:** 2024-10-15

**Authors:** Tim W.H. Rijnhout, Marin Kieft, Willemijn M. Klein, Edward C.T.H. Tan

**Affiliations:** 1 Department of Surgery, Radboud University Medical Center, Geert Grooteplein Zuid 10, Nijmegen, The Netherlands; 2 Department of Medical Imaging, Radboud University Medical Center, Nijmegen, The Netherlands

**Keywords:** Intraosseous infusion, Trauma, Resuscitation, Emergency Department

## Abstract

**Purpose:**

During resuscitation in emergency situations, establishing intravascular access is crucial for promptly initiating delivery of fluids, blood, blood products, and medications. In cases of emergency, when intravenous (IV) access proves unsuccessful, intraosseous (IO) access serves as a viable alternative. However, there is a notable lack of information concerning the frequency and efficacy of IO access in acute care settings. This study aims to assess the efficacy of intraosseous (IO) access in acute care settings, especially focusing on children in a level 1 trauma center.

**Methods:**

This retrospective study included patients with IO access presented in a level 1 trauma center emergency department (ED) between January 2015 and April 2020. Data regarding medication and fluid infusion was documented, and the clinical success rate was calculated.

**Results:**

Of the 109,548 patients that were admitted to the ED, 25,686 IV lines were inserted. Documentation of 188 patients of which 73 (38.8%) children was complete and used for analysis. In these 188 patients, a total of 232 IO accesses were placed. Overall, 182 patients had a functional IO access (204 needles) (88%). In children (age < 18 years) success rate was lower as compared to adults, 71–84% as compared to 94%. However, univariate regression showed no association between the percentage of functional IO access and gender, age, weight, health care location (prehospital and in hospital), anatomical position (tibia as compared to humerus) or type of injury.

**Conclusion:**

Intraosseous access demonstrates a high success rate for infusion, independent of gender, age, weight, anatomical positioning, or healthcare setting, with minimal complication rates. Caution is especially warranted for children under the age of six months, since success rate was lower.

**Supplementary Information:**

The online version contains supplementary material available at 10.1186/s12873-024-01103-w.

## Introduction

During resuscitation in emergency situations, establishing intravascular access is crucial for promptly initiating the delivery of fluids, blood, blood products, and medications [1]. When intravenous (IV) access fails or is insufficient, intraosseous (IO) access provides a rapid, stable, safe, and effective alternative [2, 3]. IO access was first introduced in the 1940s but disappeared due to the introduction of plastic peripheral venous needles. In the 1980s, IO access was re-introduced as a rapid alternative after the failure of peripheral venous access. Nowadays, the European Resuscitation Council Guidelines for Resuscitation recommend IO access when the first attempts at establishing IV access are unsuccessful or challenging. The challenges encountered may be influenced by patient-specific factors, such as age and weight, the expertise of healthcare practitioners, the type of cannula employed, and the anatomical location on the body [4]. In pediatric resuscitation, the use of IO access is recommended when it is likely to be difficult to obtain IV access [5]. While the utilization of IO access has been deemed suitable in resuscitation, there remains ongoing debate, with some expressing reservations regarding flow rates and the potential hemolysis of blood products [6, 7]. Notably, even in austere environments, the efficacy of IO access has been demonstrated, albeit with a recognized risk of hemolysis [8]. The success rate for placement varies from 53 to 97% in both adult and pediatric cases [9, 10]. However, detailed information regarding the use and effectiveness of IO access in emergency situations is scarce. Therefore, the aim of this study is to investigate the incidence and success rate of IO access in children and adults in acute care settings. We hypothesize that intraosseous access is a safe and effective alternative to peripheral venous access.

## Materials and methods

### Study design and setting

This is a single center retrospective cohort study of the use of IO access in the ED from 01 to 2015 to 04-2020. The study was performed in a level 1 trauma center (Radboud University Medical Center Nijmegen, the Netherlands) and was approved by the local medical ethical committee Arnhem / Nijmegen and acknowledges the standards and assessment framework of further use of patient data for research purposes (file number: 2020–6267). Obtaining informed consent was waived by the committee follow Dutch guidelines for retrospective investigation.

### Participants

All patients who were presented to the ED from 01 to 2015 to 04-2020 who had a documented IO access use, were included. We also included patients who had IO access initiated before arriving at the hospital.

### Variables

The primary outcome measure was the incidence of IO access, and the secondary outcome was the documented clinical success rate. Patient demographics, including age, weight, height, body mass index, circulatory parameters, health care location, anatomical location of the IO access insertion (tibia, humerus), documented success, and complications of the IO access were collected. Body mass index was calculated and categorized as normal, underweight, overweight, or obese according to age [11].

### Data sources / measurements

Patient data were collected from the medical records in the Electronic Patient File (EPF) EPIC^®^ (Epic Systems Corporation, 1979, Milky Way Verona). All files were screened for ED visits and the registration of an IO access using the term “IO needle” as well as synonyms such as “bone needle” and “intraosseous access.” The IO needle used in the study hospital’s helicopter emergency medical services (HEMS)/emergency medical services (EMS) area and ED is the EZ-IO System (Vidacare Corporation, San Antonio, TX, USA). The clinical position of IO needle placement was considered successful if there was documentation of aspiration and smooth infusion through the IO needle, or if it was documented that the IO needle was in a clinically sufficient position (when infusion was possible, it was defined as clinically sufficient). The authors confirm that the data supporting the findings of this study are available within the article and its supplementary materials.

### Statistical methods

The data was analyzed with SPSS (IBM SPSS statistics for Windows version 25.0, Armonk, NY, USA). Clinical success rate was calculated by dividing the cases with successful IO needle use by all IO needle use cases. The basic characteristics of the population (means, medians, and proportions, where appropriate) were determined and stratified by the functional placement of a needle. Cases were grouped by gender, age, healthcare setting, anatomical location, and presence of traumatic injury for analysis. Additionally, logistic regression was performed to examine the association between gender (male/female), age (in years), weight (in kg), patient type (trauma/non-trauma), anatomical location (tibia/humerus), placement setting (prehospital/in-hospital), and the functionality of IO access. Categorical variables were converted into dummy variables to be included in the regression model. The dependent variable, IO access outcome, was specified as a categorical variable. Odds ratios were calculated, and presented alongside 95% confidence intervals and *p*-values. *P*-value < 0.05 was considered to indicate statistical significance.

## Results

**Fig. 1:**
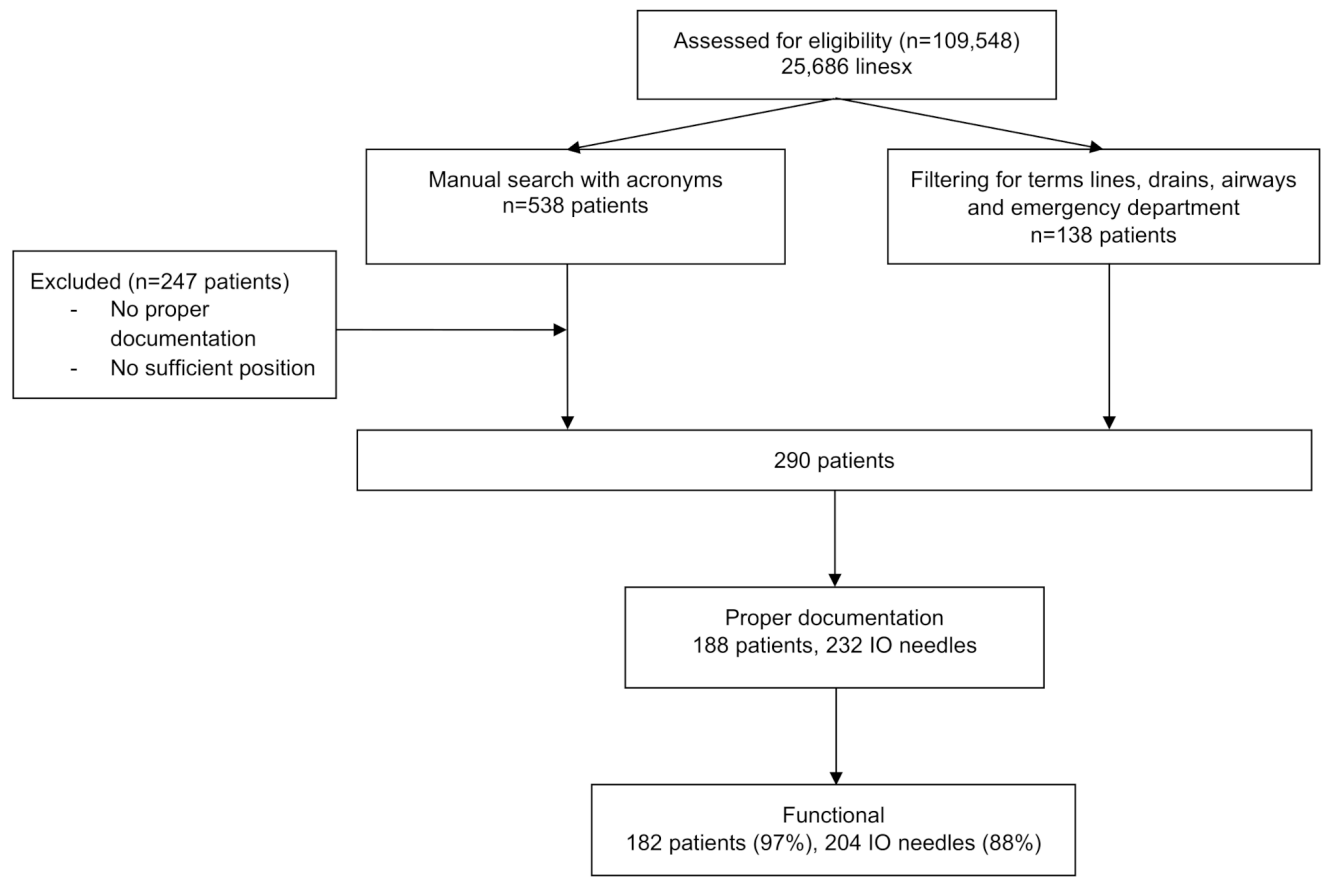
Flowchart for patient inclusion

**Fig. 2:**
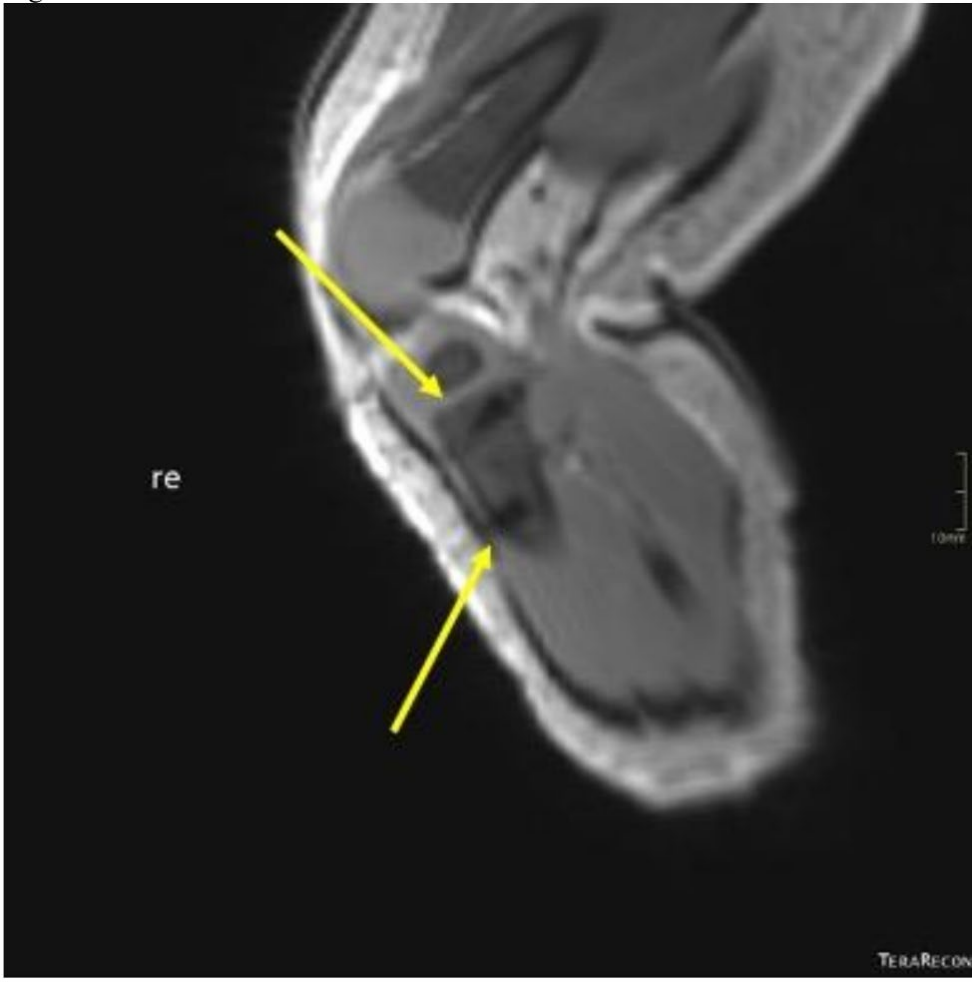
The trajectory of the IO needles (black) can be clearly seen at the end of the yellow arrows

From 2015 to 2019, 109,548 patients were admitted to the ED of this level 1 trauma center. In this level 1 trauma center, approximately 2% is severely injured with an injury severity score of > 16. In this period, a total of 25,686 IV lines were inserted. Filtering the electronic patient file for the terms lines and drains, resulted in 290 patients with documentation of IO access. Proper documentation regarding functionality was available for 188 patients of which 73 (38.8%) were children. In total, 232 needles were placed of which 204 were defined functional (overall success rate of 88%). Overall, 182 patients had a functional needle (97%). For 28 needles infusions was not possible due to failure (presumable because of incorrect position). Thirty-five patients (19%) received two needles, three cases received three needles and in one case four IO needles were placed (study flowchart in Fig. 1). In this child cardiopulmonary resuscitation case, four attempts were made in both tibia and femur (Fig. 2).

### Associations

Table 1 summarizes the clinical success rate for gender, age, weight, (anatomical) location of placement, setting (trauma / non-trauma) and percentage of functional needles. In children (aged < 18 years) success rate was lower as compared to adults (71–84% as compared to 94%).

**Table 1:** Clinical success rate for different body types and location of placement

	Functioning needle	Total	Success rate IO needle (95% CI)
Age (*n* = 232)			
< 0.5 years	15	21	71% (48–89)
0.5–2 years	27	35	77% (59–90)
2–18 years	32	38	84% (69–94)
> 18 year	130	138	94% (89–97)
Situation (*n* = 232)			
Trauma	165	182	91% (85–94)
Non-trauma	39	50	78% (64–88)
BMI (*n* = 116)			
< 18	34	43	79% (64–90)
18–20	7	10	70% (35–93)
21–25	21	21	100% (84–100)
26–30	23	23	100% (85–100)
> 30	18	19	95% (74–99)
Location (*n* = 232)			
In hospital	32	37	86% (71–95)
Prehospital	135	152	89% (83–93)
Both	17	20	85% (62–97)
Unknown	20	23	87% (66–97)
Anatomical location (*n* = 232)			
Tibia	122	142	86% (79–91)
Humerus	13	13	100% (75–100)
Both	8	13	62% (32–86)
Unknown	61	64	95% (87–99)
Infusion (*n* = 222)			
Fluid	182	201	91% (86–94)
Blood	13	13	100% (75–100)
Combination	8	8	100% (63–100)

95% CI, confidence interval

Univariate logistic regression analysis demonstrated no statistically significant associations between the outcome and the following variables: gender (OR = 0.90), age (OR = 0.98), weight (OR = 0.99), prehospital placement (OR = 1.00), anatomical location (OR = 0.81), and trauma (OR = 0.82), as all *p*-values were greater than 0.05 (Table 2).

**Table 2:** Univariate logistic regression analysis for functional needles

	Univariate		
	Odds ratio (95% CI)	*p*	*N*
Gender – Male	0.90 (0.20–4.15)	0.894	188
Age	0.98 (0.96–1.01)	0.261	188
Weight	0.99 (0.97–1.02)	0.594	131
Prehospital placed needle	1.00 (0.11–8.90)	1.00	168
Anatomic position – tibia	0.81 (0.35–1.86)	0.624	188
Trauma	2.82 (0.61–13.15)	0.186	188

CI, confidence interval

### Adverse events

In 18 of 232 (7.7%) needles, a complication was reported. Extravasation occurred in three cases (3/232, 1.3%). In four cases, the IO catheter was removed due to pain after placement. In two patients, compartment syndrome was diagnosed after needle insertion, but was not directly associated to the needle insertion. One of these two patients developed striker position foot after an IO needle placement in their leg; this was most likely caused by a hematoma in the peroneus loge that then triggered compartment syndrome. Both cases were treated with a fasciotomy. In one patient the needle was placed intra-articular and penetrating the growth plate according to an x-ray; the needle was then removed. In eight needles, wound leakage and hematoma were described, with no further long-term consequences.

## Discussion

The objective of this study was to examine the incidence and success rate of intraosseous (IO) access utilization in emergency situations within both pediatric and adult populations. The implementation of IO access was carried out in 0.3% of patients admitted to this level 1 trauma center, demonstrating a high overall clinical success rate of 97%.

The incidence of IO access placement in this cohort (2.7 per 1000 ED visits for adults and 0.9 per 1000 ED visits for pediatrics) was higher compared to other countries such as the United States (0.05 per 1000) and Japan (0.34 per 1000) [12, 13]. However, in contrast to these studies, our study only includes patients admitted to a level 1 trauma center, which makes it more likely that this cohort contains more severely injured or ill patients. For these severely injured patients, rapid and early vascular access is important and should be performed in a prehospital setting. No discernible disparity is observed between prehospital and in-hospital intraosseous (IO) access placement, as noted by Wampler et al., who reported a first attempt success rate of 91%, increasing to 94% after a second attempt [14]. Based on these results, IO access must be considered during resuscitation, including prehospital settings.

The overall success rate of 97% in this study is comparable to other scientific reports. The randomized controlled trial conducted by Reades et al. reported a success rate of 91% for IO access placement [15]. However, the clinical success rate drops in pediatric patients. We demonstrated that children aged 6 months or younger had lower success rates (71%) compared to the overall success rate. There are several explanations for these results, which are in line with the study performed by Myers et al. [16]. First, the target area of the pediatric tibial bone is small, with smaller bone shafts and largely cartilaginous epiphyses. Additionally, adults have the advantage of having a flat cortical surface along the medial aspect of the tibia, with only a thin cover of soft tissue. In an infant’s tibia, the tibial target area has a more rounded contour [17]. Besides the greater target area and larger bone size in adults, there is also a thicker cortex in the bone marrow. Because of the thinner cortex in infants, any slight movement of the IO access after entering the bone marrow would likely result in a greater chance of dislocating and failing [9]. Second, there may also be an association between needle length and failure [9]. Harcke et al. revealed that a 25-mm access was not successful in six out of seven placements in infants of 2 years of age or younger. Among patients that had a 15-mm access, 18 of 29 accesses were within the medullary cavity of the bone marrow. Harcke et al. report that the 15-mm access length has a poor success rate in small infants and advise caution in using this IO access for young infants [17]. The advised length for pediatric patients from 3 to 39 kg weight is 15 mm, 25 mm for adult patients > 39 kg weight, and 45 mm in length for obese patients (using the EZ-IO kit) [18]. It should be noted that besides the EZ-IO system, there are other IO access products available, such as the Bone Injection Gun (BIG) and the FAST1 System, which may have different success rates and usage characteristics. In contrast to other studies, body mass index was not associated with success rate. For example, Pifko et al. described a success rate of 97% in patients > 8 kg and 47% for patients < 8 kg. They equated 8 kg to an average age of 6 months; although this study grouped patients by age, comparison between the studies is nonetheless valid [9]. For neonatal patients, IO access seems even more challenging [19]. To improve the success rate of IO access use in children under the age of 6 months and patients with lower body weight, more routine IO simulation training may be beneficial [9].

This study found a 7.7% complication risk for IO access use, a lower percentage compared to IV access (23–44%) [20, 21]. However, adverse events after IO access are often severe, such as extravasation, compartment syndrome, pain, osteomyelitis, growth plate injuries, and fracture. Beyond a few case studies, no numbers are known for the incidence of compartment syndrome after using an IO access. A meta-analysis review of 4,270 cases of IO access found 27 (0.6%) cases of osteomyelitis [22]. In the present study, there was one case in which the access may have penetrated the growth plate. It has been shown that penetration of the growth plate does not result in a subsequent leg length discrepancy [23]. Although the frequency of adverse events is low, misplacement can lead to serious complications. To investigate influencing factors on the success rate, especially in infants aged < 6 months, further research should be conducted in a prospective setting.

Despite IO access being a safe and effective tool for rapid access, intravenous access remains and will be the gold standard for most cases. This is understandable since it is less invasive and burdensome for the patient. However, a randomized controlled trial (RCT) comparing IO and IV access during cardiac arrest revealed a 48% difference in first attempt success rate in favor of IO access [15]. Also, during trauma resuscitation, first attempt IO access placement was higher compared to first attempt intravenous access [3]. In this study, no separate analysis was performed for these patient groups.

### Limitations

This is a retrospective database study based on documented IO needle use. The study design may have led to underestimation of the incidence and success rate of IO needle in the ED due to the insufficient data reportion or grammatical inaccuracy. Several devices exist for IO insertion, including First Access for Shock and Trauma (FAST1), the EZ-IO, and the Bone Injection Gun (BIG). In addition, the definition of success was based on retrospective interpretation of file notes. In patients with no other infusion system mentioned beyond the IO needle, the documentation of successful injection of drugs is interpreted as a clinically successful IO needle use. The retrospective design of this study has limitations. It was not possible to extract variables such as needle length, the experience of an individual health care professional, or the time needed to insert the needle from the files, but these variables may have affected patients’ outcomes. Another limitation is non-independence due to multiple instances per patient. Since logistic regression accounts for independent observations, repeated measures could impact the accuracy. While the impact on results is likely minimal adjustments like cluster-robust standard errors or mixed-effects models were not applied. Additionally, other variables were not always documented, such as weight, blood pressure, and heart rate. It is imaginable that due to this low reportion rate, the analysis was not significant. The limited documentation could be explained by the emergent setting given that there is little time and a hectic atmosphere in the ED.

## Conclusions

Intraosseous access demonstrates a high success rate for infusion, independent of gender, age, body size, anatomical positioning, or healthcare setting, with minimal complication rates. Consequently, it represents a viable substitute for peripheral venous access during emergency resuscitation. Caution is especially warranted for children under the age of six months, as the percentage of functional needles was lower in this group.

## Electronic supplementary material

Below is the link to the electronic supplementary material.

Supplementary Material 1

## Data Availability

The authors confirm that the data supporting the findings of this study are available within the article [and/or] its supplementary materials.

## Abbreviations

BMI: Body mass index
ED: Emergency department
EPF: Electronic patient file
HEMS: Helicopter emergency medical services
IV: Intravenous
IO: Intraosseous
